# An Adrenocortical Carcinoma Evolving After Nine Years of Latency From a Small Adrenal Incidentaloma

**DOI:** 10.7759/cureus.16851

**Published:** 2021-08-03

**Authors:** Harpreet S Kohli, Sukesh Manthri, Shikha Jain, Rahul Kashyap, Sheng Chen, Thoyaja Koritala, Aysun Tekin, Ramesh Adhikari, Raghavendra Tirupathi, Aram Barbaryan, Simon Zec, Hanyin Wang, Stephanie Welle, Pavan Devulapally, Mack Sheraton, Manpreet Kaur, Vishwanath Pattan, Chaitanya K Mamillapalli, Salim R Surani, Syed Anjum Khan, Nitesh K Jain

**Affiliations:** 1 Department of Hospital Medicine, Saint Vincent Hospital, Erie, USA; 2 Department of Oncology, Mary Bird Perkins Cancer Center, Houma, USA; 3 Department of Internal Medicine, MVJ Medical College, Bengaluru, IND; 4 Department of Anesthesiology and Critical Care Medicine, Mayo Clinic, Rochester, USA; 5 Department of Pathology, Memorial Medical Center, Springfield, USA; 6 Department of Internal Medicine, Mayo Clinic Health System, Mankato, USA; 7 Department of Hospital Medicine, Franciscan Health, Lafayette, USA; 8 Department of Geriatrics, Brown University, Providence, USA; 9 Department of Internal Medicine, Keystone Health, Chambersburg, USA; 10 Department of Internal Medicine, University of Kansas Health System, Kansas City, USA; 11 Department of Critical Care Medicine, Mayo Clinic, Rochester, USA; 12 Department of Hospital Medicine, Mayo Clinic Health System, Mankato, USA; 13 Department of Cardiology, Mayo Clinic Health System, Mankato, USA; 14 Nephrology, Methodist Hospital, San Antonio, USA; 15 Emergency Medicine, Johns Hopkins University, Baltimore, USA; 16 Department of Medicine, Drishti Advanced Eye and Diabetes Care Center, Patiala, IND; 17 Division of Endocrinology, Wyoming Medical Center, Casper, USA; 18 Division of Endocrinology, Southern Illinois University School of Medicine, Springfield, USA; 19 Department of Endocrinology, Springfield Clinic, Springfield, USA; 20 Department of Pulmonary and Critical Care Medicine, Texas A&M University, Corpus Christi, USA; 21 Department of Critical Care Medicine, Mayo Clinic Health System, Mankato, USA

**Keywords:** adrenal incidentaloma, adrenal carcinoma, clinical guidelines, benign adrenal tumor, indeterminate adrenal nodule, secretory tumors of adrenal gland, adrenal disease, adrenal pheochromocytoma, adrenal disorders, adrenal surgery

## Abstract

Adrenal incidentalomas (AIs) are common incidental findings in medical practice with clinical significance. Although most AIs are nonsecretory and nonmalignant, they require a short course of follow-up over one to two years to rule out malignancy or hormonal secretion according to clinical practice guidelines. However, this can result in some adrenocortical carcinomas (ACCs) being missed if they transform at a later stage or evolve slowly. Here, we report one such case of an AI, which although remained indolent, eventually transformed into an ACC many years after the initial detection.

## Introduction

An adrenal incidentaloma (AI) is an adrenal lesion equal to or more than 1 cm in size discovered inadvertently during evaluation for reasons other than suspected adrenal pathology, usually excluding individuals with a history of cancer [1-4]. AIs are common and can be unilateral or bilateral [2,3]. Most AIs are benign nonsecretory adenomas [3-5]. The major concerns for AI include (i) developing secretory behavior (10-15%) and (ii) risk of malignancy (2-5%) [4,6]. Therefore, workup involves follow-up imaging and hormone testing, which is imperative and should be individualized [2,4]. AIs less than 1 cm in size are considered benign and not followed through [2,4].

Here, we report the case of a woman with an AI who was diagnosed to have an adrenocortical carcinoma (ACC ) many years after the initial detection and hence falling outside the purview of current guidelines.

## Case presentation

**Figure 1:**
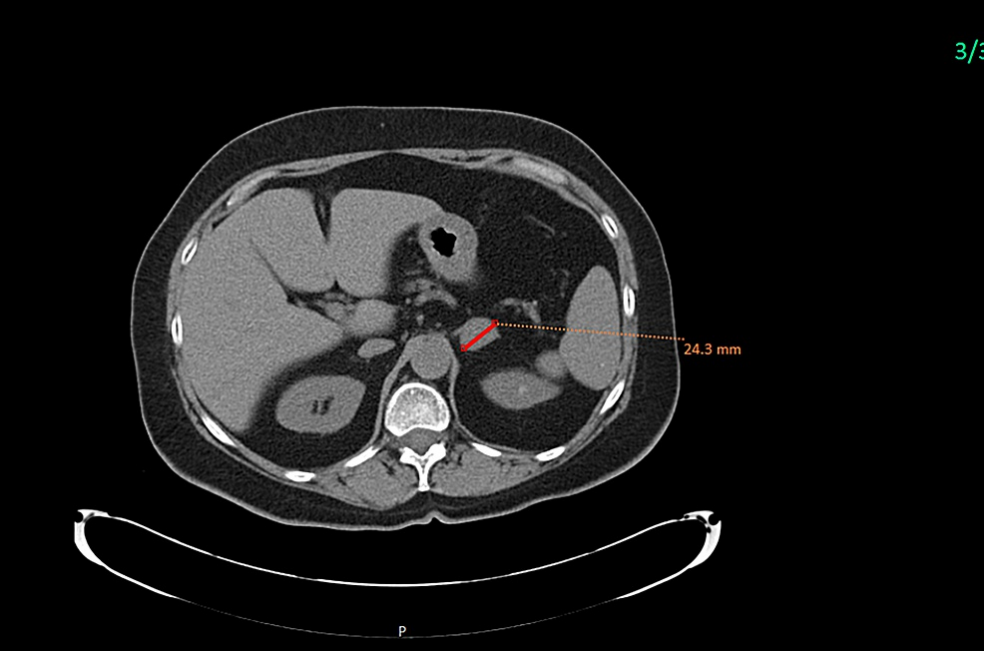
Initial CT image showing the apparent benign left adrenal incidentaloma, which is homogenous, rounded, and has a low density measuring 24.1 mm (maximum diameter). CT: computed tomography

A 70-year-old female with a history significant for essential hypertension, osteopenia, and Crohn’s disease was referred to the Endocrinology Clinic for further evaluation of a left adrenal lesion. About nine years ago, she was found to have a 2 × 1.6 cm left adrenal nodule on computed tomography (CT) scan of the abdomen (Figure 1), with enhancement greater than 10 Hounsfield units (HU) and contrast washout density consistent with adenoma (absolute contrast washout of 67%, relative washout of >47%) and indeterminate adrenal nodule. Functional workup for pheochromocytoma, hyperaldosteronism, and hypercortisolism was normal.

Subsequent surveillance CT imaging study at one year showed the stable size of the adrenal nodule. The patient had several follow-up CT scans (at years four, five, and seven) performed for other indications, which recorded the stability of the left adrenal nodule. One month prior to the current visit, the patient presented with abdominal pain and diarrhea.

On further questioning, the patient reported that her previously well-controlled hypertension with lisinopril and hydrochlorothiazide significantly worsened a month prior to the current visit requiring modification to her medication regimen. The patient also noted some increased facial hair growth which required trimming in the last few months. The physical examination was within normal limits, except for uncontrolled blood pressure.

**Figure 2:**
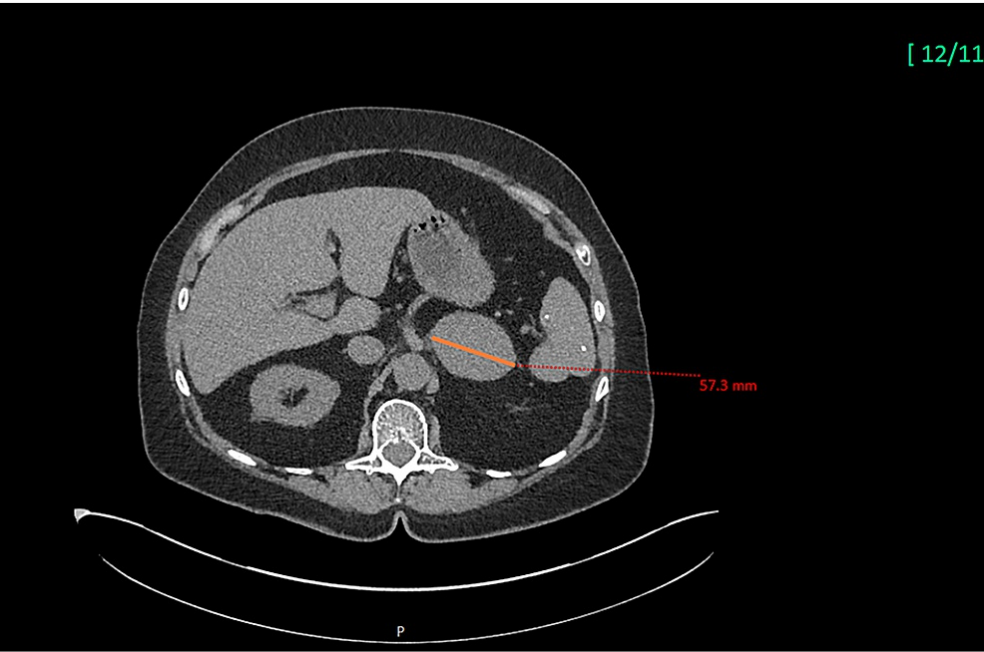
Image showing the progression of the apparently benign left adrenal incidentaloma (arrow in Figure 1) to adrenocortical carcinoma (arrow). CT scan images nine years later showing the change in the characteristics of the lesion, with a rapid interval growth measuring 57.3 mm (maximum diameter). CT: computed tomography

Repeat CT imaging (Figure 2) of the abdomen, demonstrated a significantly increased left adrenal nodule compared to a prior study (Figure 1) measuring about 5.8 × 4.4 cm (Figure 2). Precontrast HU measured 37, postcontrast HU measured 98, and delayed HU measured 62 with the image phenotype consistent with malignancy. Magnetic resonance imaging (MRI) was obtained which demonstrated an enlarging 5.8 cm left adrenal mass. Prior to this study, the nodule had remained stable for eight years.

Laboratory evaluation revealed that the 24-hour urinary catecholamines, plasma metanephrines, renin, and aldosterone levels were within normal limits. Additionally, the 1 mg dexamethasone suppression test showed an elevated cortisol level of 26 µg/dL (normal: <1.8 µg/dL), suggestive of excessive autonomous cortisol production. The patient had a long-standing history of osteopenia without any history of fractures. The dual-energy X-ray absorptiometry scan revealed that the lumbar spine T-score was -1.8 and the femur neck T-score was -1.5.

Given the rapidly growing adrenal lesion, which was concerning for malignancy, she was promptly referred to the Urology service and underwent left adrenalectomy. She was started on hydrocortisone for the management of adrenal insufficiency postoperatively. Her antihypertensive medication including hydrochlorothiazide, lisinopril, and amlodipine was discontinued as she remained normotensive during hospitalization following adrenalectomy.

**Figure 3:**
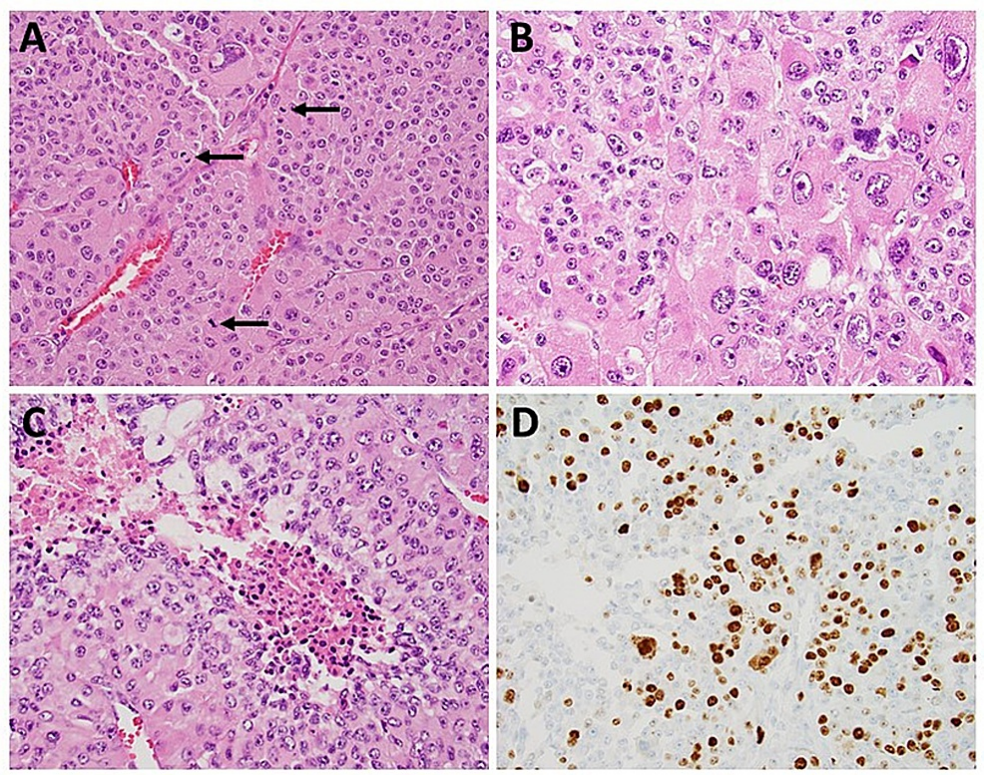
Sections of the left adrenalectomy specimen. The sections reveal a cellular neoplasm with frequent mitotic figures (indicated by arrows, A), marked nuclear atypia (B), presence of focal tumor necrosis (C), and high proliferation index by immunohistochemical stain for Ki-67 (D).

The left adrenalectomy specimen showed the presence of an 8.0 × 5.3 × 3.9 cm tumor. Sections of the tumor revealed a cellular neoplasm with frequent mitotic figures (up to 25/50 HPF, Figure 3A) with marked nuclear atypia (Figure 3B), presence of focal tumor necrosis (Figure 3C), and adrenal vein invasion. The tumor was located 0.2 mm from the closest surgical margin. Immunohistochemical stain for Ki-67 demonstrated a high proliferation index of about 20-30% (Figure 3D). The neoplastic cells were strongly positive for vimentin, synaptophysin, calretinin, inhibin, and focally positive for Melan-A. They were negative for cytokeratin AE1/3, epithelial membrane antigen, chromogranin, paired box 8, and S100. Based on these histopathological and immunohistochemical findings, a diagnosis of ACC with the tumor stage of pT2Nx was rendered.

Given the high Ki index, the patient was started on radiation treatment along with mitotane. She was also started on hydrocortisone replacement for the management of mitotane-induced adrenal insufficiency. Unfortunately, the patient developed progression of ACC with pulmonary and peritoneal metastasis and was refractory to chemotherapy. She died about 15 months after the initial presentation with ACC.

## Discussion

The term “incidentaloma” was coined in 1982 by Geelhoed and Druy. AIs are incidental lesions that come to attention when abdominal imaging is performed for symptoms and signs unrelated to adrenal disease. They are clinically unapparent adrenal masses more than 1 cm in size, and the patient is required not to have any known history of cancer [3,4].

Most commonly, AIs are benign and do not secrete any hormones, although exceptions do occur, and hence, they need to be evaluated with periodic follow-up, as failure to do so results in significant morbidity and mortality [3,4]. The prevalence of AI in autopsy series has varied from 1.05% to 8.7% [4]. Modern imaging modalities with higher resolution also report a similar prevalence of about 4.4-7.3%, with the elderly population reporting up to 10% prevalence [4,7,8]. AIs become increasingly common with advancing age, occurring unilaterally or sometimes bilaterally, and are more likely to be found on the left side as the latter could be more apparent to the radiologist [4]. Although previous studies report that it is more common in the female gender, more recent literature shows that it is slightly more common in the male gender [4,8].

The current management of AI is based on clinical practice guidelines, expert opinion, and retrospective observational data. Until recently, there was a lack of good prospective quality data to validate these recommendations [6].

Almost all AIs need to be screened for adrenal catecholamine and cortisol hypersecretion [4,8]. The only exception to this rule is adrenal tumors with a very low density (less than 10 HU) [9]. Adrenal sex hormones can be checked in patients who exhibit signs and symptoms of virilization and or gynecomastia [4,8]. Furthermore, mineralocorticoids are screened in patients with hypertension and/or hypokalemia [4,8].

Once the patient is screened to be negative biochemically, serial clinical evaluation can proceed for about five years annually. Any evidence of worsening comorbidities such as hypertension, obesity, and diabetes mellitus or clinical features of new-onset endocrine disease would warrant reconsideration of performing hormonal biochemical tests, especially for testing autonomous cortisol secretion [3,4,7]. Although the latter recommendations are consistent with the 2016 guidelines of the European Society of Endocrinology/European Network for the Study of Adrenal Tumors and the 2011 guidelines of the Italian Association of Clinical Endocrinologists, other professional societies from the United States, Canada, Korea, Poland, and France recommend biochemical testing annually for a variable period [7].

Imaging is the next line of workup. CT scan, both nonenhanced and contrast-enhanced timed washout studies, MRI chemical shift analysis, and 18-fluorodeoxyglucose positron emission tomography (FDG-PET) in combination with CT (PET-CT) are modalities that are used in clinical practice [4].

Size of the tumor is an important criterion as increased size leads to a greater probability of adrenal carcinoma: 2% risk in AIs less than 4 cm in size, 6% risk in AIs 4.1-6 cm in size, and 25% risk of carcinoma if the size is more than 6 cm [4]. A cut-off value of 4 cm gives a high sensitivity of 93% but a low specificity of 24% [4], and hence additional criteria are needed.

Conventionally, in a nonenhanced CT scan, a density cut-off of less than 10 HU helps distinguish a benign from a malignant lesion or a secretory mass like a pheochromocytoma [4]. A low-density mass is indicative of adipose tissue and hence benign with exceptions like myelolipoma which need to be considered [4].

About 30% of adrenal tumors may have a density of more than 10 HU, requiring other investigations such as adrenal CT contrast washout studies, where contrast is administered and scans are obtained twice, immediately after administrating contrast and after 10-15 minutes [4]. Although both benign and malignant adenomas enhance after contrast administration, only benign adenomas demonstrate a washout of more than 60% in the delayed scan images [4].

Chemical shift MRI scans should be used when CT scans cannot be obtained such as during pregnancy or other conditions or when sufficient ambiguity exists regarding the characteristics of the masses [4]. FDG-PET is another modality that has demonstrated high sensitivity and specificity of 91% and is most useful to confirm the presence of metastatic disease [4].

Overall, benign adenomas are characterized by the size of <4 cm, regular shape, homogeneous content, conventionally noncontrast CT attenuation less than 10 HU, and with absolute contrast washout >60%, relative washout >40% with CT contrast [3,5,10].

Once a diagnosis of AI is made, individualized risk stratification is warranted for the possibility of ACC. Nonsecretory AIs of less than 4 cm in size and homogenous density of less than 10 HU do not require any further follow-up [3,4]. An AI that does not satisfy the aforementioned criteria requires further follow-up [3,4]. The management of such lesions should ideally be directed by a multidisciplinary team consisting of an endocrinologist, a radiologist, a pathologist, and a surgeon.

As mentioned earlier, an AI more than 4 cm in size has poor specificity for malignancy, requiring follow-up in 6-12 months [3,4]. If the tumor size increases to 5 mm or above in absolute diameter with more than 20% increase compared to baseline, such growth is termed “significant” and is an indication for adrenalectomy [3,4]. A growth that does not satisfy this cut-off warrants further follow-up imaging with a CT scan in another 6-12 months [3,4].

According to a systematic review and meta-analysis encompassing 4,121 patients in follow-up, the mean tumor growth was only 2 mm over 52.8 months, with only 2.5% of AIs demonstrating growth of more than 10 mm, with no new development of adrenal cancer and clinically significant development of hormonal excess demonstrated in only less than 0.1%. This finding suggested that benign adrenal adenomas have limited growth potential and are less likely to show malignancy in the future [11].

In another study, which was performed by way of a review of published literature, clinical practice guidelines were applied to AIs in 2009. The study revealed that during follow-up as per clinical guidelines, clinically significant development of hormonal excess was demonstrated in only less than 0.1%, and malignancy was diagnosed in very few patients (0.2%). Furthermore, false-positive rates of the imaging and other studies were typically 50 times greater than true-positive rates [5]. Any strategy that includes fine-needle aspiration or needle biopsy of the adrenal gland is nonproductive and risky as it may involve such complications as seeding of malignancy along the needle track [5,12].

Hence, guideline-recommended follow-up therapy can lead to an increase in tests and procedures which may not be productive both for the patient and from a cost-benefit analysis.

An average (CT) scan follow-up exposes each patient to 23  mSv of ionizing radiation, which is equivalent to 1 in 430-2,170 chance of causing malignancy. This is similar to the possibility of developing adrenal malignancy during a three-year follow-up of AI, and hence prolonged serial CT scan follow-up cannot be recommended, as the risk-benefit profile and cost-effectiveness become unfavorable [5,7].

Hence, it follows from the above discussion that AIs of more than 4-6 cm threshold, growth of 5 mm or above in absolute diameter with more than 20% increase compared to baseline in a 6-24-month period, inhomogeneity or increased density of more than 10% HU, exhibiting excess hormone secretion can be considered for adrenalectomy. In reality, age, comorbidities, and risk-benefit of surgery versus watchful observation are weighed carefully by a multidisciplinary team and clinical judgment is applied to individual patient scenarios [7].

A few other cases [7,12], similar to our case, have been described in the literature where AI was indolent for many years but subsequently demonstrated malignancy. Such a development is rare and falls outside the purview of current guideline recommendations. Likely this could be due to slow-growing cancer or transformation from a benign to malignant form [7,12]. The latter transformation could be due to de novo idiosyncratic transformation. Such transformation is known to occur in nature and in human beings, where benign colonic adenoma can transform into a malignant tumor [7,12].

In a recently published prospective, multicentric, international study of AI involving 2,017 patients, three different characteristics, namely, tumor size (<4 cm or >4 cm), image characteristics of the adrenal tumor, and urine steroid metabolomics (mass spectrometry-based steroid metabolite profiling of 24-hour urine samples combined with machine learning-based data analysis), were studied and the results were analyzed [6]. The study showed that the increase in cut-off from 10 HU to 20 HU improved the specificity of the nonenhanced CT scan from 64% to 80% without compromising sensitivity, and hence can significantly reduce follow-up imaging and surgeries [6], which is of clinical significance.

Furthermore, urine steroid metabolomics alone had a higher positive predictive value (PPV) of 34.6% (95% confidence interval: 28.6-41.0) compared to either imaging test for tumor size 19.7% (16.2-23.5) or imaging characteristics 19.7% (16.3-23.5) [6].

The combination of tumor diameter greater than 4 cm, nonenhanced CT tumor attenuation greater than 20 HU, and urine steroid metabolomics indicating a high risk of ACC provided a PPV of 76.4% and a negative predictive value of 99.7% for ACC [6].

In summary, urine steroid metabolomics has a higher PPV than ionizing imaging tests [6], and the application of the combined triple tests to all routinely diagnosed AI can potentially reduce the number of imaging procedures required to diagnose ACC and can avoid unnecessary surgery of benign adrenal tumors, hence reducing patient morbidity and healthcare costs [6].

## Conclusions

Although rare, ACC has a poor prognosis and high mortality if the diagnosis is delayed, with a five-year survival of <15% in advanced stages. Although the surgical outcomes of ACC have vastly improved, the prognosis is poor if it is not diagnosed and treated early.

Here, we report a case of an indeterminate adrenal nodule at the time of initial diagnosis, which despite serial follow-up CT scans at four, five, and seven years showing stable adrenal nodule, had a change in behavior and size finally leading to adrenalectomy and a diagnosis of ACC. Our case highlights the limitations of current guidelines in the management of AI. In addition, it illustrates the dilemma regarding the optimal follow-up strategies in patients with indeterminate adrenal nodules, and the need for careful clinical follow-up of these patients to pursue symptoms and adrenal hormonal evaluation to detect ACC at an early stage. This case depicts the challenge in the timely diagnosis of such ACCs while preventing unwarranted surveillance of benign lesions.
